# Complex, Dynamic Combination of Physical, Chemical and Nutritional Variables Controls Spatio-Temporal Variation of Sandy Beach Community Structure

**DOI:** 10.1371/journal.pone.0023724

**Published:** 2011-08-17

**Authors:** Kelly Ortega Cisneros, Albertus J. Smit, Jürgen Laudien, David S. Schoeman

**Affiliations:** 1 Leibnitz Center for Marine Tropical Ecology, Bremen, Germany; 2 School of Biological and Conservation Sciences, University of KwaZulu-Natal, Durban, South Africa; 3 Alfred Wegener Institute for Polar and Marine Research, Bremerhaven, Germany; 4 School of Environmental Science, University of Ulster, Coleraine, Northern Ireland; National Institute of Water & Atmospheric Research, New Zealand

## Abstract

Sandy beach ecological theory states that physical features of the beach control macrobenthic community structure on all but the most dissipative beaches. However, few studies have simultaneously evaluated the relative importance of physical, chemical and biological factors as potential explanatory variables for meso-scale spatio-temporal patterns of intertidal community structure in these systems. Here, we investigate macroinfaunal community structure of a micro-tidal sandy beach that is located on an oligotrophic subtropical coast and is influenced by seasonal estuarine input. We repeatedly sampled biological and environmental variables at a series of beach transects arranged at increasing distances from the estuary mouth. Sampling took place over a period of five months, corresponding with the transition between the dry and wet season. This allowed assessment of biological-physical relationships across chemical and nutritional gradients associated with a range of estuarine inputs. Physical, chemical, and biological response variables, as well as measures of community structure, showed significant spatio-temporal patterns. In general, bivariate relationships between biological and environmental variables were rare and weak. However, multivariate correlation approaches identified a variety of environmental variables (i.e., sampling session, the C∶N ratio of particulate organic matter, dissolved inorganic nutrient concentrations, various size fractions of photopigment concentrations, salinity and, to a lesser extent, beach width and sediment kurtosis) that either alone or combined provided significant explanatory power for spatio-temporal patterns of macroinfaunal community structure. Overall, these results showed that the macrobenthic community on Mtunzini Beach was not structured primarily by physical factors, but instead by a complex and dynamic blend of nutritional, chemical and physical drivers. This emphasises the need to recognise ocean-exposed sandy beaches as functional ecosystems in their own right.

## Introduction

In most ecological systems, including those of the oceans, community structure emerges from a complex interplay between biotic interactions and abiotic environmental factors [1]–[6]. An exception to this apparent rule seems to be ocean-exposed sandy beaches. These systems are highly dynamic in space and time, and as a result, are traditionally referred to as “physically stressed” [7], in the sense that individual resident macrobenthic species appear to respond independently to physical features of the environment, with the influence of biotic interactions being negligible [8]. Although evidence to the contrary is slowly accumulating [9]–[12], the role of biotic interactions in structuring the macrobenthic communities of intertidal beaches is still considered trivial in all but the least disturbed and lowest energy systems [13].

Anthropogenic influence is nowadays ubiquitous in marine communities [14]. Of particular concern for beaches is the rate of coastal urbanisation [15], which brings with it extensive coastal armouring as well as additional elements of disturbance. These all have adverse effects on local ecology [16]–[18], and should further minimise the influence of ecological interactions [13]. Under such circumstances, greatest abundance and diversity of resident macroinfauna would be expected on fine-sand, dissipative beaches [19], while coarse-sand, reflective beaches should harbour small populations of only a few species [20].

On the other hand, much of the evidence for physical control comes from studies that measured only physical beach features [8], [20], [21]; characteristics such as primary productivity and food availability have been largely ignored, or considered as being subsumed within sampling designs. Among the few studies that have assessed chemical variables, salinity has been the most prominent and most influential in community structure [22]. Despite the importance of biological interactions (including food availability) as a factor controlling the structure and dynamics of benthic communities in general [6], [23]–[27], as well as those of other soft-sediment shores [2], [3], [28], [29], few studies of beach community ecology have taken this into account [30]–[32]. It is therefore evident that broader consideration of the roles of a range of potential structuring processes should enhance understanding of the ways in which sandy beach macrobenthic communities are structured.

Opportunities to investigate the relative roles of physical, chemical and nutritional gradients in structuring intertidal beach macrobenthic communities arise around estuary mouths. Because intertidal sandy beaches are devoid of biogenic structure and support little primary production [33], they depend heavily on allochthonous subsidies. In this sense, adjacent ecosystems, such as estuaries, can provide significant inputs of both inorganic nutrients to the surf zone and particulate organic matter to the intertidal zone [34]. Moreover, estuaries mediate strong salinity gradients, which have been demonstrated to have a detrimental influence on population and community attributes of sandy beach assemblages [22], [35]–[37]. Unfortunately, however, few integrated attempts have been made to include a wide range of physical, chemical and nutritional variables as potential explanatory variables for patterns of intertidal community structure at the meso-scale. Here, we report on a study that takes advantage of the strong seasonal changes in estuarine flow of the Mlalazi Estuary on the KwaZulu-Natal (KZN) coast of South Africa to investigate macrobenthic community structure at Mtunzini Beach in the presence and absence of various gradients associated with estuarine inputs.

**Figure 1:**
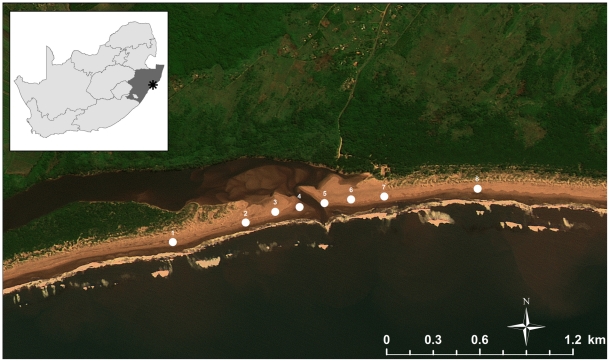
Map of the study area, indicating the position of the sample stations relative to the Mlalazi estuary. Numerals indicate station numbers (Station 1 is in the far south; Station 8 is in the far north).

KZN, on the east coast of South Africa (Fig. 1), experiences marked seasonal cycles in rainfall, resulting in strong temporal patterns of river flow to the extent that many estuaries in this region are isolated from the sea for much of the dry winter season by sandbars that block their mouths [38]–[40]. Because KZN marine waters are reasonably oligotrophic [41], seasonal freshwater inflow represents the main source of nutrients for estuarine food webs [42]–[46]. During the rainy season, estuarine flow increases in volume; consequently, larger proportions of estuarine seston and nekton are exported to coastal waters and adjacent systems, including sandy beaches [47]. Due to this cycle, estuarine input to Mtunzini Beach should be minimal toward the end of a dry season, with any depression in salinity or elevation in nutrient concentrations and associated surf-zone plankton productivity being limited to the immediate vicinity of the estuary mouth. If the beach macroinfauna are sensitive to estuarine inputs, there might be aggregation at (or avoidance of) areas immediately adjacent to the estuary mouth, with the communities beyond the reach of these putative gradients distributed along the shore according to physical conditions, with higher macrofaunal abundance and diversity on sections of the beach with fine sand and wide intertidal zones. By contrast, as rainfall increases and the river starts to fill, estuarine input to the surf zone should increase and any resulting effect should spread along the shore. Any corresponding response by the macroinfaunal communities is likely to be detectable as consistent relationships with chemical or nutritional variables and would provide evidence that responses to other variables can override the effect of physical beach descriptors.

By sampling an along-shore grid of stations on Mtunzini Beach at three discrete times that correspond to the end of the dry season, the start of the rainy season and the late rainy season, this study aims to determine whether the beach community responds overwhelmingly to the physical environment or if other factors are influential. To address this aim, the objectives are: (1) to determine if alongshore patterns for physical, chemical and nutritional features of the beach vary through time; (2) to determine whether alongshore patterns in intertidal macrobenthic community structure vary through time; and (3) to determine, which physical, chemical and nutritional variables are most influential in describing changes in macrobenthic community structure.

## Methods

The Mlalazi Estuary (28°58′S; 31°48′E, eastern coast of South Africa, Fig. 1) is a permanently open estuary with its mouth closing only during major droughts. The catchment of approximately 492 km^2^ [48] is subject to high rainfall of about 1250 mm.yr^−1^, peaking in austral summer (December–February). The estuary length extends from about 11 km in the dry season to 7 km during the rainy season [49]. The microtidal Mtunzini Beach (maximum tidal range is 2.13 m) borders the estuary and stretches about 5 km both north and south of the estuary mouth. Nel and Bezuidenhout [50] classified the grain size of Mtunzini Beach as medium-coarse and documented a well-sorted sediment and an intermediate-reflective beach morphodynamic state.

Biological samples and environmental data were collected bimonthly between October 2008 and February 2009 (three sessions). Sampling was conducted around low tide within four days of a spring tide. Sampling stations were arranged symmetrically around the mouth of the Mlalazi Estuary, with four stations to the north and four to the south (Fig. 1). In each direction, stations were positioned at 25 m, 150 m, 400 m, and 900 m from the mouth.

### Ethics statement

A sampling permit for scientific investigation was issued to the School of Biological and Conservation Sciences, University of KwaZulu-Natal, by the Department of Environmental Affairs and Tourism, Republic of South Africa. Permit reference numbers were V1/1/5/1 and RES2008/14 for 2008 and 2009, respectively.

### Sampling design for macrobenthos

At each station, biological sampling consisted of triplicate shore-normal transects at 10-m intervals alongshore. Each transect was sampled at ten across-shore sampling levels, arranged at uniform intervals between the drift line and the spring low-water mark. At each across-shore level, four sediment samples including macroinfauna were excavated using a stainless steel corer of 18-cm diameter (surface area of 0.0254 m^2^) inserted to a depth of about 30 cm. These four samples were pooled and washed through a 1-mm nylon-mesh sieving bag in the swash zone to remove excess sand. All materials remaining in the sieve bag were stored in labelled, sealed polyethylene bags, frozen on return to the laboratory and stored at −20°C until analysis [51], [52]. This field sampling design largely conforms to recommendations by Schlacher et al. [53] and represents a sampling effort of 3.05 m^2^ per station and session. Although this sampling effort was smaller than that recommended by Schoeman et al. [54], [55], our aim was to assess spatial and temporal trends in community structure on the basis of the most common species, rather than to accurately estimate macroinfaunal species richness or abundance. In this sense, the ability to sample more stations compensates for the slight loss in accuracy and precision of biotic measures at individual stations caused by smaller-than-ideal sample sizes.

### Sampling design for environmental variables

For the measurement of physico-chemical variables (Table S1), triplicate water samples were collected below the water surface (ca. 5–10-cm depth) in the swash zone of the eight sandy beach stations, and in the mouth of the estuary during each session. Separate triplicate samples were taken for the determination of photopigment concentrations, organic matter content and dissolved inorganic nutrient concentrations.

For photopigment concentrations, 250-ml water samples were collected in acid-washed plastic bottles from each station. These samples were cooled on ice and stored in the dark for a maximum of two hours before further processing. For the determination of size-fractioned photopigment concentrations, the samples were serially filtered (vacuum, <50 kPa) through 20-µm Nitex mesh (for microplankton photopigments), 2-µm membrane filters (for nanoplankton photopigments), and 0.72-µm GF/F filters (for picoplankton photopigments), and then extracted in 90% acetone for 24 hrs in the dark at 4°C. Photopigment concentrations were determined using a Turner Trilogy fluorometer (Sunnyvale, California, US) before and after acidification with two drops of 1% HCl [56].

Corresponding 1-l water samples for the determination of Total Suspended Solids (TSS) and Particulate Organic Matter (POM) were collected in plastic bottles at each station, and were treated identically to the photopigment samples prior to processing. In the laboratory, the water was filtered (vacuum, <50 kPa) through pre-combusted and pre-weighed GF/F filters. The filters were then dried at 60°C for 24 hrs, reweighed and the amount of TSS determined by mass difference. Additionally, POM determinations were made from these filters after combusting them at 450°C for 12 hrs before reweighing. POM was estimated by mass difference before and after combustion.

For the determination of Particulate Organic Carbon (POC), Particulate Organic Nitrogen (PON) and C∶N ratio, water samples were collected, stored and processed as described above for TSS determinations. GF/F filters, on which POM had been collected were acidified with 2N HCl and again dried at 60°C for 24 hrs. POC and PON were determined using a Flash EA 1112 series elemental analyzer (Thermo Finnigan, Milan, Italy). Results were expressed as percentages of carbon and nitrogen.

For the quantification of Dissolved Inorganic Nitrogen (DIN: nitrate+nitrite) and Dissolved Inorganic Phosphorus (DIP: ortho-phosphate) concentrations, 50 ml of water filtrate from the photopigment determination (after filtering through GF/F filters) were collected in acid-washed bottles and frozen at −20°C. Nutrient concentrations were determined using a Skalar San++ continuous-flow analyser (Skalar Analytica BV, The Netherlands), following the methods of Mostert [57]. Salinity measurements were determined from the resulting filtrate in the laboratory using a hand-held refractometer (ATAGO, Japan).

Sediment samples for the determination of the granulometric measures and Sediment Organic Matter (SOM) content were taken using a sediment core (internal diameter of 2 cm) to 10-cm depth at each shore level and station. To determine SOM, 5 g of oven-dried (60°C for 24 hrs) sediment was weighed and combusted at 450°C for six hours before reweighing. Organic matter content was determined as the difference in sediment masses. At each station, SOM content was determined as the mean value of the ten samples collected. For grain size analysis, the remaining dried sand was passed through nested sieves with a standard range of φ intervals [58], and conventional statistics derived [59].

### Macrobenthic community analysis

After defrosting the material collected from the sieved macrofauna cores, organisms were separated from the remaining sediment by manual elutriation: the sample was placed in a 25-l plastic bucket containing 10 l of distilled water, and stirred vigorously; thereafter, the water was poured through a 1-mm mesh size sieve. This procedure was repeated five times. Govender [60] determined that three to four repetitions of elutriation are required to extract 95% of all macrofaunal organisms from sediment samples taken at nearby KZN beaches, and that five repetitions almost always extract all such organisms. Following elutriation, the remaining sediment was searched manually for large, heavy-bodied organisms, such as clams and whelks. Specimens were identified to the lowest taxonomic level possible and enumerated.

### Data analysis

Granulometric parameters (mean grain size, sorting, kurtosis and skewness) were estimated with the software GRADISTAT Version 4.0 [61]. Beach morphodynamic state was classified according to Dean's dimensionless fall velocity (Ω) [62], [63]. Sand-fall velocities were obtained from granulometric determinations; wave height and period data were obtained from the WindGuru website (http://www.windguru.cz, accessed on June 13^th^, 2009).

Species abundance was estimated as the number of individuals per strip transect (IST) [53], [64], [65]. Only beach-resident species were considered, and because we intended to conduct a range of multivariate analyses, all species that occurred in fewer than five transects (out of a total of 72 transects) were excluded from the analysis, to reduce the number of zeros in the data matrix. Data were fourth-root transformed (to further equalize the contributions to community structure by common and rare species) before the construction of a Bray-Curtis similarity matrix [66] to represent resemblances in community structure among transects from each station and session.

Inspection of correlograms of environmental variables revealed significant positive skew in many variables as well as several nonlinear relationships among variables. To ameliorate potential problems in subsequent analyses, all variables were transformed by their natural logarithm (ln(*x*+1)) to linearise relationships and to provide more symmetrical distributions. All physical variables were then converted to *z*-scores (i.e., scaled to a mean of zero and unit variance) to avoid scale dependence in subsequent analyses. Potential collinearity among environmental variables was explored by inspecting the correlogram of the transformed data and by calculating condition indices of the principal components of the correlation matrix, as recommended by Quinn and Keough [67].

Hypotheses regarding spatio-temporal relationships were tested using Pearson correlation, while those concerning patterns were tested using PERMANOVA, as described by Anderson et al. [68]. In the latter case, session, side (north/south of the estuary mouth) and station (nested within side) were considered to be fixed factors. Following each PERMANOVA, *post-hoc* tests were used to further explore significant interactions or main effects, using Monte Carlo approximate *p*-values [68] where insufficient unique permutations existed for meaningful tests. Where multivariate hypotheses were tested, non-Metric Multidimensional Scaling (nMDS) was used to illustrate relationships among samples [66].

Because environmental data were collected on a per-station basis, rather than on a per-transect basis, all assessments of relationships between physico-chemical variables and community structure were made on per-station basis. To determine the set of physical variables that best described the observed spatio-temporal structure in the community data, the Envfit and Bioenv functions of the Vegan package in R were used [69]. The Envfit function fits individual environmental variables onto a given nMDS ordination of biotic variables in such a way as to maximize the correlation between the two data sources. By contrast, the Bioenv function investigates different combinations of environmental variables in an attempt to identify that subset for which the Euclidean distance resemblance matrix provides the maximum Spearman correlation with the Bray-Curtis community resemblance matrix. For the Envfit analyses, session and side were treated as factors (categorical variables) and the remaining variables as vectors (continuous variables). For the Bioenv analysis, we initially considered all environmental variables, including session and side (which had to be treated as continuous variables in this context). The procedure was then rerun without either session or side to assess whether some suite of other environmental variables might provide a solution to explain similar amounts of the variance.

In multivariate analysis (Bioenv), we are aware of no criteria that penalize improvement in fit relative to the number of parameters (as would be done in a more conventional model fitting by the Akaike Information Criterion, for example). We therefore set a simple *a priori* criterion for the “optimal” environmental matrix for the Bioenv analysis as that with the smallest number of variables, which improves the correlation by at least 10% relative to the strongest single-factor correlation. Although we acknowledge that this criterion is arbitrary, we feel that beyond this point, each additional environmental variable adds relatively little to the explanation of the ordination, and might potentially introduce spurious correlations.

Because we conducted many statistical tests, the probability of falsely rejecting at least one null hypothesis (i.e., the probability of a false positive result) would be substantially larger than the conventionally chosen α-level of 0.05 [70]. Although the sequential Bonferroni test is sometimes used to adjust inflated α-levels on a case-by-case basis, there are several mathematical, logical and practical objections to its use [71], [72]. We therefore acknowledge that there is a strong possibility that some of our hypothesis tests will falsely reject true null hypotheses and thus set α to 0.01 to ameliorate this risk. While this concession appears arbitrary and will not eliminate false positives, it does provide a practical and consistent criterion for hypothesis tests and reduces the false positive rate by a factor of five.

Data manipulation and most analyses were carried out in R [73]; PERMANOVA and verification of multivariate analyses were conducted using PRIMER 6 [74].

## Results

### Physical environment

**Figure 2:**
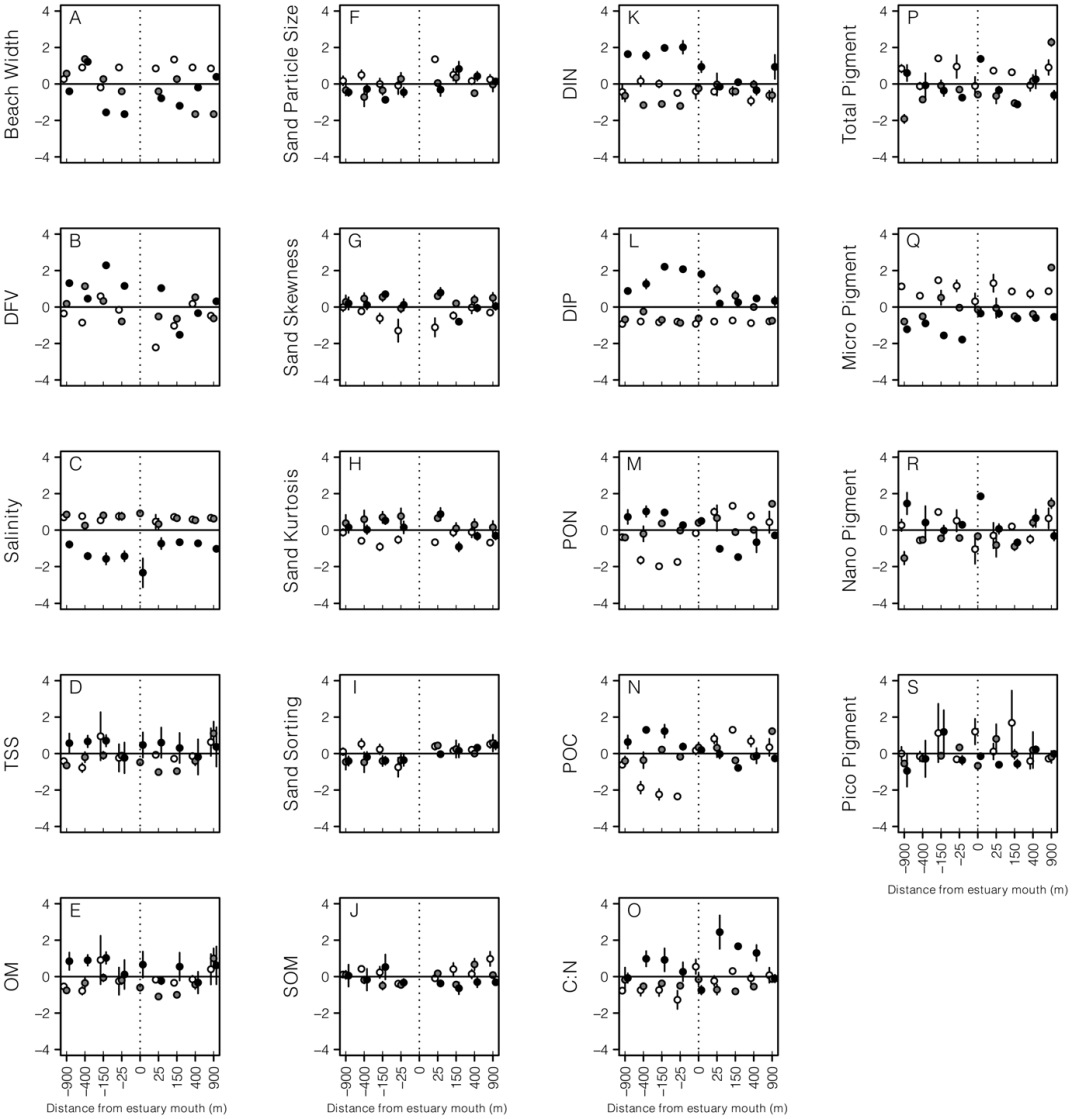
Plots of physical variables collected at the sample stations on Mtunzini Beach. All data are log-transformed and standardized to a mean of zero and SD of 1. Samples from October 2008 are indicated with white circles, those from December 2008 with grey circles and those from February 2009 with black circles. Error bars are standard deviations. For clarity, the *x*-axes are not plotted to scale. Negative distances are stations to the south of the estuarine mouth. Mean and standard deviation of untransformed data are: (A) Beach width (67.44±14.71 m), (B) DFV (3.69±0.52), (C) Salinity (33.25±5.89), (D) TSS (52.92±19.02 mg.l^−1^), (E) OM (42.97±17.23 mg.l^−1^), (F) Sand Particle Size (428.68±101.32 µm), (G) Sand Skewness (1.66±0.71), (H) Sand Kurtosis (8.29±4.31), (I) Sand Sorting (218.44±92.32), (J) SOM (1.29±1.05%), (K) DIN (0.62±0.65 µM), (L) DIP (0.9±1.21 µM), (M) PON (5.47±2.72%), (N) POC (45.79±24.11%), (O) C∶N (8.44±2.27), (P) Total Photopigment concentrations (3.52±1.10 µg.l^−1^), (Q) Microplankton Photopigment concentrations (1.08±0.62 µg.l^−1^), (R) Nanoplankton Photopigment concentrations (1.95±0.87 µg.l^−1^), (S) Picoplankton Photopigment concentrations (0.54±0.31 µg.l^−1^).

The environment along Mtunzini Beach, as described by beach morphodynamics and sediment characteristics, varied substantially in space and time (Fig. 2; Table 1, 2). The intertidal beach face fluctuated in width between 45 m and 90 m, being significantly (*p*<0.01) wider in October 2008 than in subsequent sessions (Fig. 2A; Table 1). Mean particle size showed that the sediment of Mtunzini Beach is coarse, moderately well sorted sand. Spatially, sediments were finer and better sorted south of the estuary than to the north, especially during February 2009 (Fig. 2F–I; Table 1, 2). Temporally, sediments during October 2008 were coarser and more coarse skewed than during other sessions, although skewness decreased with distance from the estuary mouth (Fig. 2F–I; Table 1, 2). According to Dean's dimensionless fall velocity (DFV), Mtunzini Beach was intermediate (mean Ω = 3.75), with no spatial or temporal patterns in morphodynamic state, besides a tendency for the beach to be more reflective during October 2008 than during the other sessions (Fig. 2B; Table 1).

**Table 1:** Significant sources of variation in univariate descriptors of the environment on Mtunzini Beach.

Category	Type	Variable	Sources of variation
Physical	Morphodynamic	Beach width	Se
		DFV	Se
Physical	Sediment	Sand particle size	Se, Si
	characteristics	Sediment skewness	Se
		Sediment kurtosis	Se
		Sediment sorting	Si
Chemical	Water chemistry	Salinity	Se×Si, Se
Chemical	Nutrients	DIN concentration	Se×Si, Se, Si
		DIP concentration	Se×St (Si), Se×Si, St (Si), Se, Si
Nutritional	Photopigments concentrations	Micoplankton photopigment concentration	Se×St (Si), Se×Si, St(Si), Se, Si
		Nanoplankton photopigment concentration	Se×St (Si), Se×Si
		Picoplankton photopigment concentration	None
		Total photopigment concentration	Se×St(Si), Se×Si, St(Si), Se
Nutritional	Organic content	SOM	None
		POM	None
		POC	Se×St (Si), Se×Si, Se, Si
		PON	Se×St(Si), Se×Si, Se, Si
		C∶N	Se, Si
		TSS	None

Significant (*p*≤0.01) results as identified by two-way fixed-effects PERMANOVA (Table S2). Abbreviations are as follows: Se = session; Si = side; St(Si) = station nested within side; and St = station (only for variables that were estimated without replication at the station level).

**Table 2:** Spatial trends in biotic and abiotic variables along Mtunzini beach during the study period.

Variable	Session	Trend	*r*	df	*P*
Sand particle size	February 2009	S-N	0.30	72	8.85×10^−3^
Sand skewness	October 2008	Away from mouth	0.30	77	6.88×10^−3^
Sand sorting	February 2009	S-N	0.36	72	1.56×10^−3^
Sand sorting	All	S-N	0.22	222	8.38×10^−4^
POC	December 2008	S-N	0.58	21	3.40×10^−3^
POC	February 2009	S-N	−0.55	22	5.84×10^−3^
PON	December 2008	S-N	0.61	21	1.93×10^−3^
C∶N	February 2009	Away from mouth	−0.52	22	9.15×10^−3^
Micro pigment	December 2008	S-N	0.69	21	2.40×10^−4^
Nano pigment	December 2008	S-N	0.78	21	5.05×10^−6^
Total pigment	December 2008	S-N	0.85	21	2.00×10^−7^
Species richness	February 2009	S-N	0.60	22	2.09×10^−3^

Pearson correlations against distance from south-most station (Trend = S-N) or absolute distance from estuary mouth (Trend = Away from mouth). Tests were run on data from individual sessions, and for all sessions combined. Only significant (*p*≤0.01) results are tabulated. Descriptions of variables and their abbreviations are listed in Table S1.

### Chemical environment

Monthly rainfall varied from 45.4 mm in December 2008 to 184.5 mm in February 2009 (Fig. S1). Instead of increasing gradually through the study period, as one may predict, monthly rainfall was relatively uniform from October to December 2008, with an increase in January 2009. Salinity measurements matched this pattern (Fig. 2C; Table 1), being similar during October and December 2008, when they were uniform alongshore, but salinity fell significantly in February 2009 (Fig. 2C; Table 1). During this final session, salinity was significantly lower south of the estuary than to the north, and was lowest in the estuary mouth (Fig. 2C). Spatio-temporal patterns in DIN and DIP concentrations reflected those in salinity, tending to be high where salinity was low (Fig. 2K, L; Fig. S1; Table 1). However, maximum concentrations were not recorded in the estuary mouth, and additional complexities were evident. Nevertheless, DIN and DIP concentrations tended to be higher during the latter sessions of the study, and this effect was more marked to the south of the estuary than to the north.

POC and PON concentrations exhibited very similar patterns and varied strongly between sides and sessions (Fig. 2M, N; Table 1). Concentrations of both differed significantly by session to the south of the estuary, with values increasing steadily through the sample period. To the north of the estuary the trend reversed, with both POC and PON concentrations significantly lower during February 2009 than during preceding sessions. This resulted in reversing spatial gradients along the beach, with increasing south-north concentration gradients in both POC and PON during December 2008, but a decreasing gradient in POC concentration during February 2009 (Table 2).

C∶N ratios were slightly lower to the south of the estuary than to the north, and were similar in October and December 2008, but were significantly higher in February 2009 (Fig. 2O; Table 1). In October and December 2008, values measured in the estuary were among the highest along the beach, whereas in February 2009, estuarine values were lowest (Fig. 2O). Despite this, C∶N ratios at beach stations decreased with distance from the estuary during February 2009 (Fig. 2O; Table 2).

The average concentrations of photopigments in the nanoplankton size range were almost twice those of the microplankton fraction, which in turn were twice those of in the picoplankton fraction (Fig. 2). Photopigment concentrations in the microplankton fraction decreased significantly through the study period on both sides of the estuary mouth (Fig. 2Q; Table 1, 2). Corresponding patterns for the nanoplankton fraction were less clear, and no trends were found for the picoplankton fraction; as a result, temporal patterns for total photopigment concentrations were inconsistent through time (Fig. 2R, S; Table 1). During December 2008, both microplankton and nanoplankton photopigment concentrations increased from south to north across the beach, a pattern reflected by total photopigment concentrations (Fig. 2P–R; Table 1, 2). In February 2009, this gradient broke down, but microplankton photopigment concentrations remained lower to the south of the estuary than to the north. Interestingly, the highest nanoplankton photopigment concentrations were recorded in the estuary mouth during February 2009.

Neither TSS nor POM concentrations exhibited any significant spatio-temporal variation (Fig. 2D, E; Table 1). Concentrations of SOM were similarly devoid of clear patterns (Fig. 2J; Table 1).

The correlogram of the scaled, transformed environmental variables (Fig. S2), indicated few potentially collinear environmental variables besides sand grain size and DFV (*r* = −0.94), PON and POC (*r* = 0.94), and OM and TSS (*r* = 0.89). This was confirmed by analysis of the condition indices of the principal components of the correlation matrix, of which only two were >30. Because the number of potentially collinear explanatory variables was small, we did not exclude any of them from subsequent analyses, but exercised appropriate care when interpreting results.

### Macroinfaunal community structure

**Figure 3:**
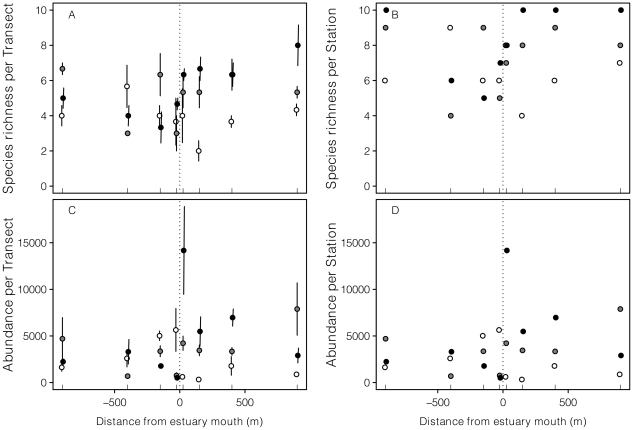
Plots of univariate measures of community structure per transect (A, C; mean ± SD) and aggregated by station (B, D). Samples from October 2008 are indicated with white circles, those from December 2008 with grey circles and those from February 2009 with black circles.

Trends in univariate measures of community structure were similar irrespective of whether they were viewed on a per-transect or per-station basis (Fig. 3). PERMANOVA and correlation analysis suggest that macroinfaunal abundance was lowest during October 2008, when both abundance and species richness were higher to the south of the estuary than to the north (Fig. 3; Table 2). By December 2008, these patterns had broken down, and by February 2009, very high numbers of recruiting mole crabs (*Emerita austroafricana*) and surf clams (*Donax madagascariensis*) immediately north of the estuary resulted in higher macroinfaunal abundance on the northern side of the beach than to the south.

**Figure 4:**
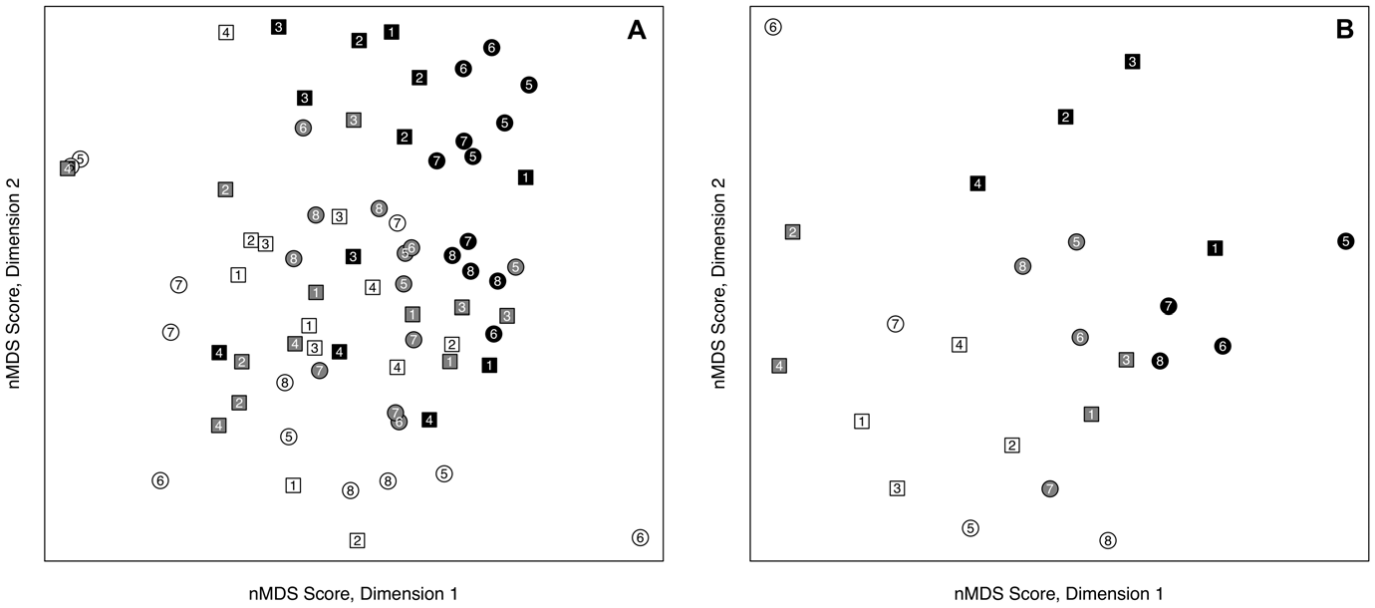
nMDS ordination of species abundance per transect (A) and aggregated by station (B), fourth-root-transformed using Bray-Curtis resemblance. Numerals indicate station numbers (Station 1 is in the far south; Station 8 is in the far north, as per Fig. 1); colors indicate session (white = October 2008; grey = December 2008; black = February 2009); and symbols indicate side of beach relative to the estuary mouth (square = south; circle = north). Stress = 0.25.

The spatio-temporal patterns in univariate community data were reflected in the multivariate community structure (Bray-Curtis resemblance on fourth-root transformed abundance data). The nMDS biplot (Fig. 4A) illustrates changes in the arrangement of the community structure on Mtunzini Beach from October 2008 to February 2009, with samples from December 2008 showing an intermediate position on the ordination between those of the first and the last sessions. The stress value for the two-dimensional ordination was relatively high at 0.25, but the significant side×session interaction detected by PERMANOVA on these multivariate data (Table 3) nevertheless reveals significant spatio-temporal patterns. Specifically, although the community structure to the south of the estuary did not differ significantly between October and December 2008, all other pair-wise comparisons were significant. Similarly, the community structure to the north of the estuary differed significantly between all sessions. The strongest (and only significant) north-south difference in community structure was detected in February 2009. Together, these results mirror the univariate analysis in suggesting a graded change in community structure through time, with strongest spatial patterns in February 2009.

**Table 3:** Significant sources of variation in univariate and multivariate measures of community structure.

Type	Response variable	Sources of variation
Univariate	Species richness	Se×Si, Se
	Macrofaunal abundance	Se×St (Si), Se×Si, Se, Si
Multivariate	Bray-Curtis resemblance by transect	Se×St (Si), Se×Si, Se, Si
	Bray-Curtis resemblance by station	Se

Significant (*p*≤0.01) results as identified by two-way fixed-effects PERMANOVA (Table S3). Abbreviations as per Table 1.

When pooling the samples by station (to accommodate comparison with environmental data, which were not measured at a per-transect resolution), replication and therefore power are lost from the PERMANOVA. Nevertheless, the nMDS ordination of this modified data set (Fig. 4B) broadly reflected that constructed on data from individual transects (Fig. 4A). The corresponding PERMANOVA (Table 3) detected a significant main effect of session, although due to reduced power, the community structure was found to differ significantly only between October 2008 and February 2009 (the *p*-value for the pair-wise test on differences in community structure between December 2008 and February 2009 was 0.019; although we considered this non significant due to our adjustment in α to account for multiple hypothesis tests, this value is nevertheless low, considering the reduced power of this test).

### Correlations between community structure and environmental variables

Correlations between univariate measures of community structure and the physical beach environment did not reveal any significant relationships when data from all sessions were considered simultaneously. The strongest (non-significant) relationships in this context were between species richness and picoplankton photopigment concentrations (*r* = 0.49, df = 22, *p* = 1.39×10^−2^) and between macroinfaunal abundance and the C∶N ratio (*r* = 0.50, df = 22, *p* = 2.05×10^−2^). Neither of these relationships were significant for individual sessions; nor were any of the observed relationships consistent across sessions. During October 2008, macroinfaunal abundance was inversely correlated with POC (*r* = −0.90, df = 6, *p* = 2.05×10^−3^) and PON concentrations (*r* = −0.88, df = 6, *p* = 3.60×10^−3^). During December 2008, there were no significant relationships and, during February 2009, species richness and abundance were positively correlated with salinity (*r* = 0.87, df = 6, *p* = 4.83×10^−3^) and C∶N (*r* = 0.85, df = 6, *p* = 8.28×10^−3^), respectively.

Inspecting the explanatory power of individual environmental variables, the Envfit routine identified four environmental vectors and one environmental factor as significant (Table 4). Salinity provided the strongest correlation, followed by DIP concentration, C∶N ratio and microplankton photopigment concentration. Session was the significant environmental factor.

**Table 4:** Fits of environmental vectors and factors to the biotic ordination of macroinfaunal community structure.

Variable	Correlation (*r*)	*p*
*Vector*		
Salinity	0.738	3.4×10^−4^
DIP	0.737	3.6×10^−4^
C∶N	0.719	4.8×10^−4^
Microplankton photopigments	0.600	9.7×10^−3^
*Factor*		
Session	0.593	6.0×10^−4^

Environmental vectors are continuous variables and factors are discrete variables. Correlations indicate the strength of the gradient in individual environmental variables across the biotic ordination. Only significant (*p*≤0.01) results are tabulated.

When using the function Bioenv to seek matrices of environmental variables that best correlate with the biotic ordination, correlations tended to increase with increasing numbers of environmental variables (Table 5). When session and side were included as explanatory variables, correlations peaked at seven variables (*r* = 0.495, this correlation before rounding was fractionally higher than that for the six-variable ordination). When excluding session and side, correlation peaked at six variables (*r* = 0.475, Table 5). When considering the relative increase in correlation with each additional variable (Table 5), the set of environmental variables identified as optimal by our *a priori* rule includes only three variables, irrespective of whether we include session and side as variables (*r* = 0.443), or not (*r* = 0.440). Permutation tests reveal that all of the selected correlations are significant (*p*<0.01).

**Table 5:** Correlations between environmental and biotic ordinations for data from Mtunzini Beach.

	Analyses including Side and Session as environmental variables		Analyses excluding Side and Session as environmental variables	
No.	Variables	*r*	Variables	*r*
1	Session	0.307	Pico pigments	0.251
2	Session, Pico pigments	0.411	C∶N, Pico pigments	0.371
3	*Session, C∶N, Pico pigments*	*0.443*	*DIP*, *C∶N*, *Pico pigments*	*0.440*
4	Session, DIN, C∶N, Pico pigments	0.463	Kurtosis, DIP, C∶N, Pico pigments	0.456
5	Session, Beach width, DIN, C∶N, Pico pigments	0.483	Beach width, DIN, DIP, C∶N, Pico pigments	0.464
6	Session, Beach width, Kurtosis, DIN, C∶N, Pico pigments	0.495	**Beach width, Kurtosis, DIN, DIP, C∶N, Pico pigments**	**0.475**
7	**Session, Beach width, Kurtosis, DIN, DIP, C∶N, Pico pigments**	**0.495**	Beach width, Kurtosis, DIN, DIP, C∶N, Pico pigment, Micro pigments	0.457

Correlations are listed with increasing numbers of variables. Left-hand panel includes factors session and side as variables, whereas the right-hand panel excludes them. Combinations of variables that maximize the correlation between biotic and abiotic ordinations are in bold, while optimal combinations of variables are italicized. Descriptions of variables and their abbreviations are listed in Table S1.

To summarize, the most important variables explaining biotic community structure are: session (identified by PERMANOVA, Envfit and Bioenv); the C∶N ratio (identified by Envfit and Bioenv, and it also had the strongest, though non-significant, correlation with macroinfaunal abundance over the study period), picoplankton photopigment concentrations (identified by Bioenv, and it also had the strongest, though non-significant, correlation with species richness over the study period), and DIP concentration (identified by Envfit and Bioenv). To these, we can tentatively add salinity, which was identified as influential only by Envfit, but did have strong correlations with macroinfaunal abundance during February 2009, when salinity values were lowest and spatial patterns in community structure were strongest.

**Figure 5:**
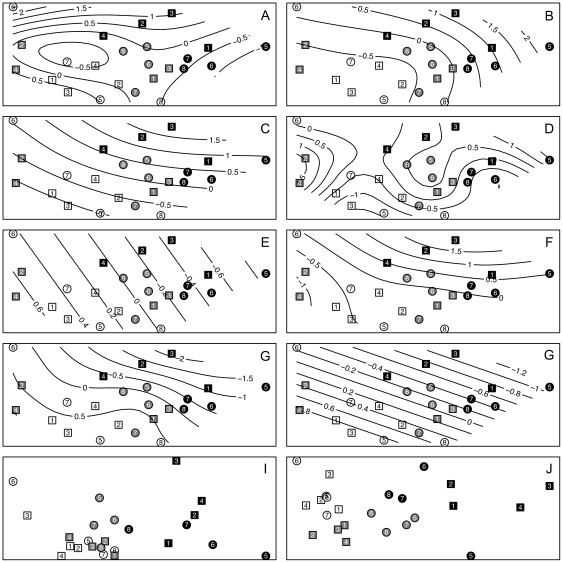
Bray-Curtis ordination of fourth-root transformed biotic variables overlaid with 2-dimensional thin-plate splines fitted by General Additive Modeling. A. Picoplankton photopigment concentrations. B. C∶N ratio. C. DIP concentration. D. Sediment kurtosis. E. Beach width. F. DIN concentration. G. Salinity. H. Microplankton photopigment concentrations. I. nMDS ordination of “optimal” set of environmental variables. J. nMDS ordination of the set of environmental variables providing the best biotic-environmental correlation. A-F are the variables identified by the Bioenv routine in order of importance (i.e. the order in which they were selected). G & H are variables selected by Envfit, but that were not selected by Bioenv. I & J are Euclidean-distance nMDS ordinations of the matrices of environmental variables for the optimal and best solutions identified by Bioenv.

Using 2-dimensional thin-plate splines fitted by General Additive Modeling, we overlaid the distribution of these selected environmental variables on the biotic ordinations (Fig. 5A–H). The results provide evidence that most of the influential environmental variables are non-linearly distributed across the biotic ordination. They also demonstrate that the variables selected first by the Bioenv routine explain outliers in the biotic ordination, and then more general patterns. For example, picoplankton photopigment concentration (the first variable selected by Bioenv) was unusually high at Station 6 in December 2008 (Fig. 2S) and the C∶N ratio (the second variable selected) was especially high at Station 5 in February 2009 (Fig. 2O). Since corresponding community samples provided outliers in the biotic ordination, and the C∶N ratio also provides some discrimination among sessions (especially separating samples from February 2009 from those of other sessions), it is not surprising that these variables together provide an ordination that is fairly strongly correlated (*r* = 0.371, Table 5) with the biotic ordination. DIP concentration adds to the abiotic ordination's ability to discriminate among all sessions; it also highlights the differences between the northern and southern sides of the beach during February 2009, when salinities were lowest and biotic patterns were strongest. The remaining variables add slowly to an increasingly complex pattern. Importantly, the nMDS biplots of both the optimal and the best explanatory environmental matrices (omitting session) showed a strong resemblance to that for the biotic variables (Fig. 5I, J). Although sessions and sides were grouped in a very similar way in both biotic and abiotic ordinations, individual stations were somewhat displaced relative to one another, reflecting the correlations of 0.440 and 0.475, respectively (Table 5).

## Discussion

The concept of overriding physical control in harsh environments is one of three paradigms describing macro-scale community patterns on exposed sandy beaches [75]. Biological factors (including food availability, competition and predation), by contrast, have been considered to play a role in structuring macrofaunal communities at the meso-scale only in physically benign habitats, such as undisturbed dissipative beaches [13]. In this study, we aim to determine if the macroinfaunal community inhabiting a physically dynamic beach (intermediate morphodynamic type) really does respond overwhelmingly to the physical environment at the meso-scale, as predicted by theory. The study site, Mtunzini Beach, is located on an oligotrophic, subtropical coast, and is bisected by the Mlalazi Estuary, which is considered the main source of nutrient inputs for the beach and surf zone. Our univariate and multivariate analyses demonstrate significant spatio-temporal variation in the physical and chemical features of the beach-surf-zone system, and also in its resident macroinfaunal community. If community structure on this beach were subject primarily to physical control, we would expect variables associated with the physical beach environment to consistently provide the best description of macrofaunal distribution through time and space.

In terms of the physical beach environment, macrobenthic abundance and species richness are expected to increase with beach width [21] and morphodynamic state (DFV) [13], and to decrease with sand particle size [20]. All of these physical variables differed significantly between October 2008 and subsequent sessions, but none exhibited significant spatial trends during either October or December 2008. Although univariate measures of community structure were similarly devoid of spatial pattern during December 2008, both macrofaunal abundance and species richness were lower to the south of the estuary than to the north during October 2008, when both biotic variables attained their minimum values and the beach was widest. This provides an initial indication that even when risks of competition and predation are most limited (i.e. when the densities of organisms are lowest) [11], [13], [76], factors other than physical characteristics of the beach can and do influence the spatial distribution of resident communities. Further evidence against the paradigm of physical control at the meso-scale is provided by the results from February 2009. During this session, the finest and best sorted sediments were found to the south of the estuary, but contrary to expectation, macrofaunal abundance was higher to the north of the estuary, and there was also a south-north increase in species richness. Our results therefore strongly suggest that physical factors are not adequate predictors of macroinfaunal abundance and species richness on Mtunzini Beach.

In terms of the chemical and nutritional composition of the surf-zone waters, significant spatio-temporal variations were observed for most variables and, as predicted, most of these patterns were associated with increased estuarine flow in February 2009. Estuarine input can directly influence macrobenthic community structure through two mechanisms. The first is a negative impact of low salinity on alongshore distribution patterns, abundance, biomass and life-history traits of sandy beach organisms [35]–[37]. The second is an increase in food resources, either through increased surf-zone primary production resulting from elevated concentrations of dissolved inorganic nutrients [34], [77], or through increased inputs of particulate organic matter [34]. During our study, salinity showed no along-shore pattern during the dry season (October and December 2008), but was lower to the south of the estuary than to the north during the rainy season (February 2009), when salinity values were lowest overall. As anticipated, estuarine input to the surf zone strongly influenced patterns in the concentrations of dissolved inorganic nutrients (DIN and DIP). However, although surf-zone phytoplankton was dominated by nanoplankton (measured by photopigment concentrations), as is common in these waters [78]–[80], elevated nutrient concentrations in the surf-zone did not boost phytoplankton concentrations. This suggests either that the residence time of DIN and DIP in the surf-zone is too short to allow phytoplankton to fully respond, or that the reduced salinity inhibited surf-zone productivity [81], [82]. Concentrations of particulate organic material were measured in our study by SOM and by swash-zone TSS, POM, POC and PON, with the C∶N ratio providing an indication of the provenance of organic matter [83]. Of these variables, POM, TSS and SOM failed to show significant spatio-temporal patterns and were unrelated to salinity or inorganic nutrient concentrations. This suggests that estuarine inputs of these materials are unlikely to directly control community structure on Mtunzini Beach. PON and POC patterns provided more contrast, and peaked to the south of the estuary during February 2009, corresponding with the presence of a plume of low-salinity estuarine water (although to the north of the estuary, both variables were at their lowest during this session). C∶N ratios also reflect a trend of increasing estuarine provenance of swash-zone organics throughout the study period, and in February 2009, when the C∶N ratio was highest, there was a strong gradient of decreasing C∶N ratio away from the estuary mouth reinforcing the notion that particulate organics were of estuarine rather than marine origin (although again, we could not account for the low C∶N measured in the estuary mouth). Despite these anomalies, on a per-session basis, relationships between univariate measures of macroinfaunal community structure and descriptors of the chemical composition of the surf-zone waters were consistently stronger than those with physical beach variables. Further, when considering all data from the study simultaneously, the strongest (although not significant at α = 0.01) biotic-abiotic relationships were trends of increasing species richness with picoplankton photopigment concentrations and of increasing macroinfaunal abundance with C∶N ratio. Together, these results confirm that physical features of the beach face are not the most influential drivers of macroinfaunal community structure on Mtunzini Beach.

Using multivariate techniques to explore the covariation in ordinations of biotic and abiotic variables, the graded change in community structure between October 2008 and February 2009, and the increasing degree of spatial structure in February 2009 were best explained by sample session, the C∶N ratio of organic matter, picoplankton photopigment concentration and DIP concentration. Sample session probably explains variability in community structure introduced by seasonal patterns such as recruitment dynamics. The other influential variables all describe features of the surf zone that are linked with food availability, either realized (particulate organics of estuarine origin, and picoplankton abundance) or potential (elevated inorganic nutrient concentrations of estuarine origin). Although we tentatively identified salinity as an additional influential variable, it is likely that most of its explanatory power derives from its strongly seasonal temporal pattern, which is already accounted for by sample session. Irrespective of the mechanisms by which the most influential variables structure the macrobenthic community of Mtunzini Beach, none of these variables describe physical aspects of the beach face. Even when increasing the number of explanatory variables beyond the “optimal” set to include all of those identified as contributing to the maximum correlation with the biotic ordination, beach width and sediment kurtosis are the only physical variables added. While macroinfaunal abundance and diversity are predicted to increase with beach width [21], the opposite appears to have occurred here, with lower abundance and fewer species present on the beach during October 2008, when the beach was widest. It is difficult to envisage a mechanism by which sediment kurtosis could influence community structure on beaches; it is therefore not surprising that kurtosis has not previously been identified as being important in this role. Results of multivariate analyses therefore support those of univariate analyses in contradicting the paradigm of physical control of community structure on Mtunzini Beach.

The present results do not contest the fact that patterns in physical processes explain significant amounts of variability in macroinfaunal species richness and abundance among geographic regions at macro-ecological scales [13], [20], [21]. They do, nevertheless, demonstrate that over smaller spatial scales, other processes are capable of driving the spatio-temporal distribution patterns of beach macroinfauna, even on physically dynamic beaches. This emphasises the fact that beaches are functional ecosystems [53], [84] that have more in common with other coastal soft-sediment systems than is generally appreciated. This is important for many reasons, not least because beaches are increasingly threatened by accelerating coastal squeeze that is driven by climate change and coastal urbanisation [85], [86]. The paradigm of physical control has encouraged coastal managers to consider the ecology of beaches subordinate to their sedimentary regime, but here we show that the ecology of individual beaches is considerably more complex. The challenge ahead lies in further exploring this complexity and in using resultant knowledge to ensure the persistence of beaches as ecosystems within the broader coastal zone.

## Supporting Information

Figure S1 **Plot of rainfall data in the catchment of the Mlalazi Estuary.** Data corresponds to station 478, Empangeni - South African Sugarcane Research Institute (SASRI).
(TIF)

Figure S2 **Correlogram illustrating the relationships among environmental variables.** Variables are arranged according to the strength of relationships expressed in a corresponding PCA of all variables. Upper triangular panel contains pair-wise scatter plots for variables. Lower triangular panel contains concentration ellipses (1 SD, mean centered) for the bivariate data, with loess-smoothed curves of the relationships. Straight lines and narrow, diagonal ellipses indicate potential collinearities.
(TIF)

Table S1 **List of the environmental variables analyzed in this study.**
(DOCX)

Table S2 **Two-way fixed-effects PERMANOVA on univariate descriptors of the physical, chemical and nutritional environment on Mtunzini Beach.**
(DOCX)

Table S3 **Two-way fixed-effects PERMANOVA on univariate and multivariate measures of community structure.**
(DOCX)
