# Supplementary figures and images for: Complex, Dynamic Combination of Physical, Chemical and Nutritional Variables Controls Spatio-Temporal Variation of Sandy Beach Community Structure
Source: PLoS One. 2011 Aug 17;6(8):e23724. doi: 10.1371/journal.pone.0023724 (PMC3157432; doi:10.1371/journal.pone.0023724)

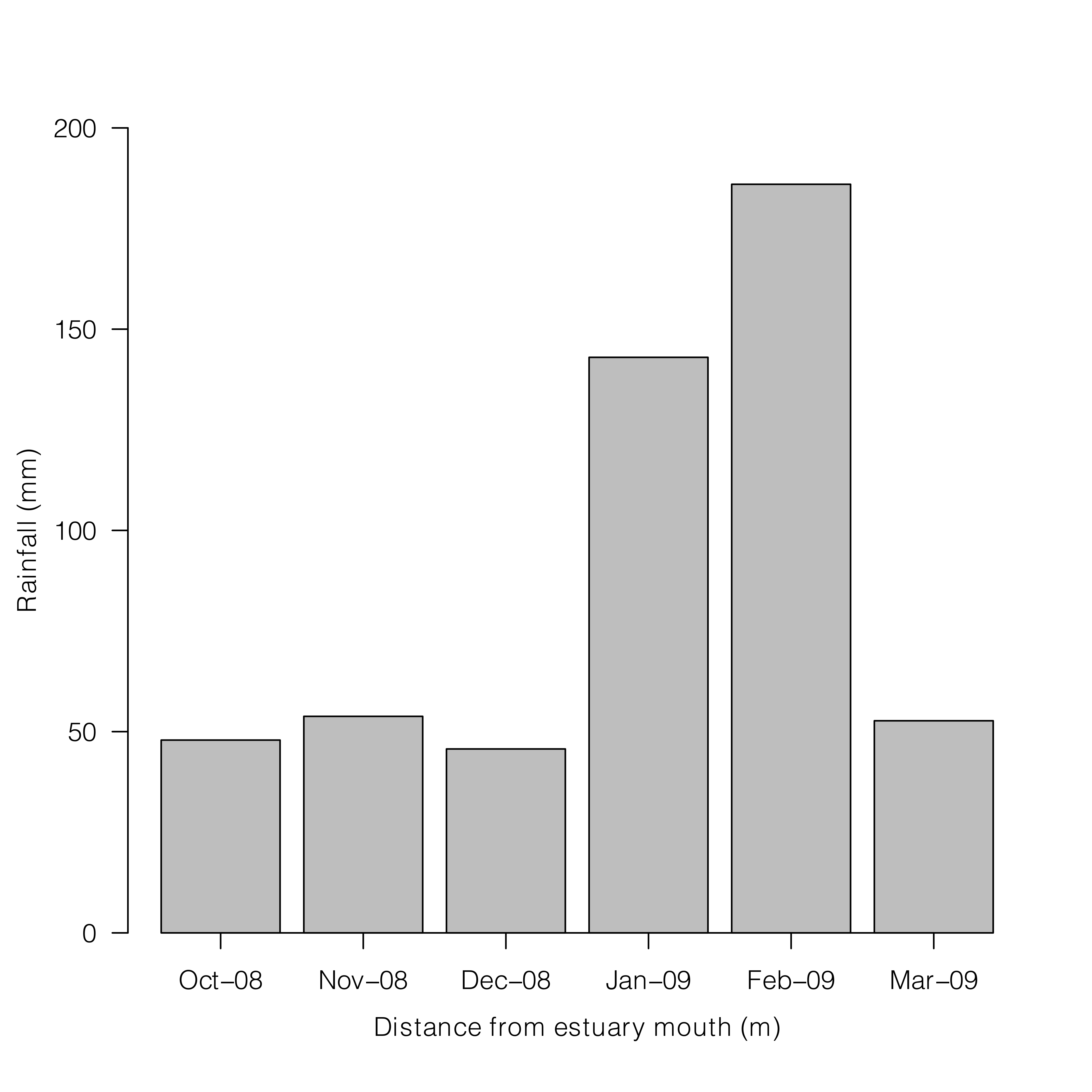

Supplement: Figure S1 — Plot of rainfall data in the catchment of the Mlalazi Estuary. Data corresponds to station 478, Empangeni - South African Sugarcane Research Institute (SASRI). (TIF) [file pone.0023724.s001.tif]

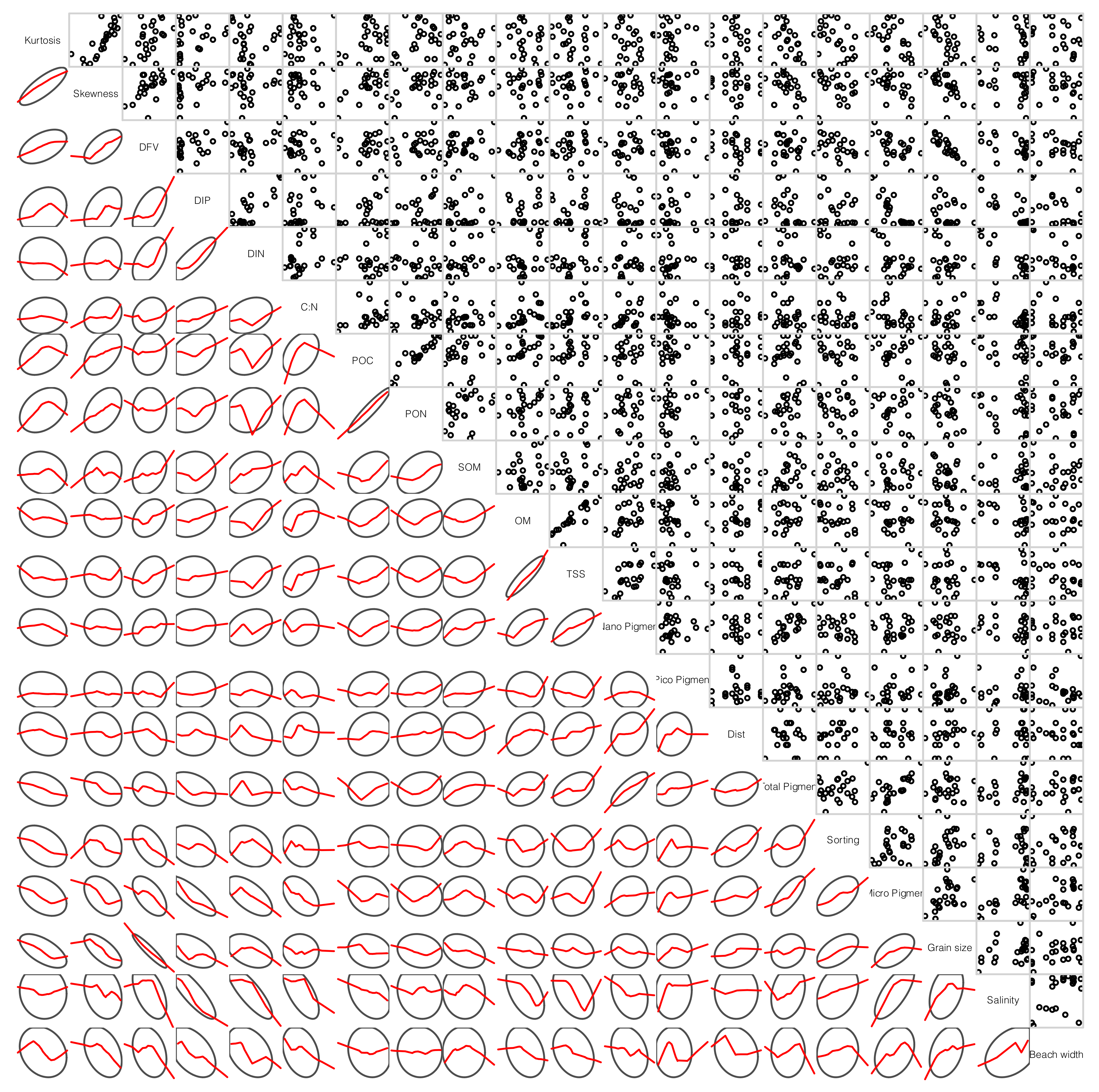

Supplement: Figure S2 — Correlogram illustrating the relationships among environmental variables. Variables are arranged according to the strength of relationships expressed in a corresponding PCA of all variables. Upper triangular panel contains pair-wise scatter plots for variables. Lower triangular panel contains concentration ellipses (1 SD, mean centered) for the bivariate data, with loess-smoothed curves of the relationships. Straight lines and narrow, diagonal ellipses indicate potential collinearities. (TIF) [file pone.0023724.s002.tif]
