# Supplementary material for: Complex, Dynamic Combination of Physical, Chemical and Nutritional Variables Controls Spatio-Temporal Variation of Sandy Beach Community Structure
Source: PLoS One. 2011 Aug 17;6(8):e23724. doi: 10.1371/journal.pone.0023724 (PMC3157432; doi:10.1371/journal.pone.0023724)
Supplement: Table S1 — List of the environmental variables analyzed in this study. (DOCX) [file pone.0023724.s003.docx]

Table S1. List of the environmental variables analyzed in this study.

| Category | Type | Variable | Description |
| --- | --- | --- | --- |
| Physical | Physical proximity | Distance to the estuary (m) | Measures proximity to the estuary mouth and therefore likely degree of influence by estuarine input. |
| Physical | Morphodynamic | Dean’s dimensionless fall velocity (DFV) | Conventionally taken as a strong predictor of community structure [1]. It ranges from reflective (narrow and steep) to dissipative Systems (wide and flat) [2]. |
|  |  | Beach width (m) | The amount of space available for habitation by intertidal macroinfauna, associated with habitat availability and suitability [3]. |
| Physical | Sediment characteristics | Mean sand particle size (µm) | One of the three most important controlling factors for sandy beach macrobenthic community and a constituent of DFV index [4]. |
|  |  | Sediment skewness | Indicates the symmetry of the sediment particle size distribution. Smaller values indicate distributions skewed towards fine particles. |
|  |  | Sediment sorting | Reflects the range of different particle size present in the sediment. Values > 2 mean poorly sorted sediments (a wide range of particle sizes). |
|  |  | Sediment kurtosis | Measure of the spread of the distribution curve. Small values indicate very tight distribution near the mean, but not necessarily in the tails |
| Chemical | Water chemistry | Salinity | Main controlling factor of alongshore distribution patterns, abundance, biomass and life-history traits of sandy beach organisms in environments influenced by a freshwater discharge [5,6]. |
| Chemical | Nutrients | Dissolved Inorganic Nitrogen (µM) (DIN)  Dissolved Inorganic Phosphorus (µM) (DIP) | Most important nutrient elements in surf-zone food webs [7] |
| Nutritional | Photopigment concentrations | Microplankton Photopigments (µg.l^-1^ ) (Micro pigments)  Nanoplankton Photopigments (µg.l^-1^) (Nano pigments)  Pico-plankton Photopigments (µg.l^-1^) (Pico pigments) | Proxy for phytoplankton biomass [8-10], which is an important controlling factor of the community structure of sandy beach consumers [11-13]. |
| Nutritional | Organic content | Organic matter in the sediment (%) (SOM)  Organic matter in the swash water (mg. l^-1^) (POM) | Proxy for detritus, which is a common and relatively constant food source for beach macrofauna [7]. |
|  |  | Particulate Organic Carbon (%) (POC)  Particulate Organic Nitrogen (%) (PON) | Food source for bacteria, which is the base of the interstitial food web in sandy beaches [7] |
|  |  | Carbon:Nitrogen ratio (C:N) | Indicator of origin of organic matter [14] Higher values are indicative of terrestrial origin. |
|  |  | Total suspended solids in the swash water (mg. l^-1^) (TSS) | Indicator of total amount of particulate matter in water, including inorganic sources. |

References

1. McLachlan A (1990) Dissipative beaches and macrofauna communities on exposed intertidal sands. J Coast Res 6: 57–71.

2. Short AD (1996) The role of wave height, slope, tide range and embaymentisation in beach classification: a review. Rev Chil Hist Nat 69: 589–604.

3. McLachlan A, Dorvlo A (2007): Species area relationships for sandy beach macrobenthos in the context of intertidal width. Oceanologia 49: 1–15.

4. McLachlan A (1983) Sandy beach ecology: a review. In: McLachlan A, Erasmus T, editors. Sandy beaches as ecosystems . W. Junk, The Hague. pp. 321–380.

5. Bergamino L, Muniz P, Defeo O (2008) Effects of a freshwater canal discharge on polychaete assemblages inhabiting an exposed sandy beach in Uruguay. Ecol Indic 9: 584–587.

6. Celentano E, Gutiérrez N, Defeo O (2010) Effects of morphodynamic and estuarine gradients on a sandy beach mole crab demography and distribution: implications for source sink habitat dynamics. Mar Ecol Prog Ser 398: 193–205.

7. McLachlan A, Brown AC (2006) The ecology of sandy shores. Burlington, MA, USA: Aca­demic Press. 373 p.

8. Valiela I (1984) Marine Ecological Processes. Springer Verlag, New York. 546 p.

9. Smith L (1996) Ecology and Field Biology. Harper Collins Publishers, New York. 740 p.

10. Menge BA, Daley BA, Wheeler PA, Strub PT (1997) Rocky intertidal oceanography: an association between community structure and nearshore phytoplancton concentration. Limnol Oceanogr 42: 57–66.

11. Lastra M, de la Huz R, Sánchez-Mata AG, Rodil IF, Aerts K, et al (2006) Ecology of exposed sandy beaches in northern Spain: environmental factors controlling macrofauna communities. J Sea Res 55: 128–140.

12. McLachlan A, Lewin J (1981) Observations on surf diatom blooms along the coasts of South Africa. Bot Mar 24: 553–557.

13. Plante-Cuny MR, Bodoy A (1987) Biomass and primary production of phytoplankton and microphytobenthos in a sheltered and an exposed beach (Gulf of Fos, France). Oceanol Acta 10: 223–237.

14. Lercari D, Defeo O (2003) Variation of a sandy beach macrobenthic community along a human-induced environmental gradient. Estuar Coast Shelf Sci 58: 17–24.
