# Supplementary material for: Complex, Dynamic Combination of Physical, Chemical and Nutritional Variables Controls Spatio-Temporal Variation of Sandy Beach Community Structure
Source: PLoS One. 2011 Aug 17;6(8):e23724. doi: 10.1371/journal.pone.0023724 (PMC3157432; doi:10.1371/journal.pone.0023724)
Supplement: Table S2 — Two-way fixed-effects PERMANOVA on univariate descriptors of the physical, chemical and nutritional environment on Mtunzini Beach. (DOCX) [file pone.0023724.s004.docx]

Table S2. Two-way fixed-effects PERMANOVA on univariate descriptors of the physical, chemical and nutritional environment on Mtunzini Beach.

|  | **df** | **Pseudo-*F*** | ***p*** | **Perm** |  | **df** | **Pseudo-*F*** | ***P*** | **Perm** |  | **df** | **Pseudo-*F*** | ***p*** | **Perm** |  | **df** | **Pseudo-*F*** | | ***p*** | | **Perm** |
| --- | --- | --- | --- | --- | --- | --- | --- | --- | --- | --- | --- | --- | --- | --- | --- | --- | --- | --- | --- | --- | --- |
| *Beach width* |  |  |  |  | *DFV* |  |  |  |  | *Sand-particle size* | | | | | *Sediment skewness* | | | | | | |
| St | 7 | 1.47 | 0.173 | 999 | St | 7 | 1.47 | 0.177 | 999 | Se | 2 | 6.51 | 0.007 | 999 | Se | 2 | 15.55 | | 0.001 | | 998 |
| Se | 2 | 9.93 | 0.001 | 999 | Se | 2 | 9.93 | 0.001 | 999 | Si | 1 | 11.62 | 0.003 | 998 | Si | 1 | 0.07 | | 0.802 | | 998 |
| Residual | 14 |  |  |  | Residual | 14 |  |  |  | St(Si) | 6 | 1.09 | 0.369 | 999 | St(Si) | 6 | 2.16 | | 0.054 | | 998 |
| Total | 23 |  |  |  | Total | 23 |  |  |  | Se×Si | 2 | 1.23 | 0.282 | 999 | Se×Si | 2 | 0.91 | | 0.383 | | 997 |
|  |  |  |  |  |  |  |  |  |  | Se×St(Si) | 12 | 2.08 | 0.023 | 999 | Se×St(Si) | 12 | 2.04 | | 0.027 | | 999 |
|  |  |  |  |  |  |  |  |  |  | Residual | 200 |  |  |  | Residual | 199 |  | |  | |  |
|  |  |  |  |  |  |  |  |  |  | Total | 223 |  |  |  | Total | 222 |  | |  | |  |
| *Sediment sorting* | | | | | *Sediment kurtosis* | | | | | *Salinity* | | | | | *DIN* | | | | | | |
| Se | 2 | 1.25 | 0.246 | 999 | Se | 2 | 14.15 | 0.001 | 999 | Se | 2 | 233.09 | 0.001 | 999 | Se | 2 | 75.98 | | 0.001 | | 998 |
| Si | 1 | 12.45 | 0.001 | 998 | Si | 1 | 1.70 | 0.190 | 997 | Si | 1 | 1.93 | 0.156 | 998 | Si | 1 | 14.21 | | 0.001 | | 997 |
| St(Si) | 6 | 0.92 | 0.451 | 998 | St(Si) | 6 | 0.63 | 0.670 | 999 | St(Si) | 6 | 1.66 | 0.144 | 999 | St(Si) | 6 | 0.46 | | 0.836 | | 998 |
| Se×Si | 2 | 0.78 | 0.440 | 998 | Se×Si | 2 | 1.72 | 0.177 | 999 | Se×Si | 2 | 8.07 | 0.001 | 998 | Se×Si | 2 | 37.08 | | 0.001 | | 998 |
| Se×St(Si) | 12 | 0.68 | 0.754 | 999 | Se×St(Si) | 12 | 1.80 | 0.063 | 999 | Se×St(Si) | 12 | 1.21 | 0.296 | 997 | Se×St(Si) | 12 | 1.93 | | 0.056 | | 999 |
| Residual | 200 |  |  |  | Residual | 200 |  |  |  | Residual | 47 |  |  |  | Residual | 47 |  | |  | |  |
| Total | 223 |  |  |  | Total | 223 |  |  |  | Total | 70 |  |  |  | Total | 70 |  | |  | |  |
| *DIP* |  |  |  |  | *POC* |  |  |  |  | *PON* |  |  |  |  | *C:N ratio* |  |  | |  | |  |
| Se | 2 | 331.67 | 0.001 | 999 | Se | 2 | 16.71 | 0.001 | 999 | Se | 2 | 6.26 | 0.004 | 997 | Se | 2 | 34.26 | | 0.001 | | 999 |
| Si | 1 | 6.09 | 0.010 | 999 | Si | 1 | 28.49 | 0.001 | 996 | Si | 1 | 14.34 | 0.002 | 998 | Si | 1 | 10.55 | | 0.002 | | 999 |
| St(Si) | 6 | 7.31 | 0.001 | 999 | St(Si) | 6 | 1.81 | 0.117 | 998 | St(Si) | 6 | 2.06 | 0.065 | 999 | St(Si) | 6 | 1.01 | | 0.416 | | 999 |
| Se×Si | 2 | 119.86 | 0.001 | 999 | Se×Si | 2 | 92.48 | 0.001 | 999 | Se×Si | 2 | 101.48 | 0.001 | 999 | Se×Si | 2 | 4.46 | | 0.016 | | 998 |
| Se×St(Si) | 12 | 10.12 | 0.001 | 999 | Se×St(Si) | 12 | 4.84 | 0.001 | 999 | Se×St(Si) | 12 | 4.30 | 0.001 | 999 | Se×St(Si) | 12 | 2.31 | | 0.018 | | 999 |
| Residual | 47 |  |  |  | Residual | 45 |  |  |  | Residual | 44 |  |  |  | Residual | 46 |  | |  | |  |
| Total | 70 |  |  |  | Total | 68 |  |  |  | Total | 67 |  |  |  | Total | 69 |  | |  | |  |
| *Microplankton photopigments* | | | | | *Nanoplankton photopigments* | | | | | *Picoplankton photopigments* | | | | | *Total photopigments* | | | | | | |
| Se | 2 | 187.99 | 0.001 | 999 | Se | 2 | 4.56 | 0.015 | 999 | Se | 2 | 0.87 | 0.404 | 999 | Se | 2 | 18.09 | | 0.001 | | 998 |
| Si | 1 | 23.07 | 0.001 | 998 | Si | 1 | 0.05 | 0.814 | 994 | Si | 1 | 0.19 | 0.680 | 996 | Si | 1 | 1.14 | | 0.298 | | 997 |
| St(Si) | 6 | 8.57 | 0.001 | 999 | St(Si) | 6 | 2.16 | 0.072 | 998 | St(Si) | 6 | 1.33 | 0.253 | 999 | St(Si) | 6 | 4.85 | | 0.001 | | 999 |
| Se×Si | 2 | 12.34 | 0.001 | 998 | Se×Si | 2 | 6.16 | 0.009 | 999 | Se×Si | 2 | 0.35 | 0.712 | 999 | Se×Si | 2 | 9.26 | | 0.001 | | 999 |
| Se×St(Si) | 12 | 9.91 | 0.001 | 999 | Se×St(Si) | 12 | 2.86 | 0.008 | 999 | Se×St(Si) | 12 | 1.13 | 0.395 | 999 | Se×St(Si) | 12 | 6.51 | | 0.001 | | 999 |
| Residual | 45 |  |  |  | Residual | 44 |  |  |  | Residual | 46 |  |  |  | Residual | 43 |  | |  | |  |
| Total | 68 |  |  |  | Total | 67 |  |  |  | Total | 69 |  |  |  | Total | 66 |  | |  | |  |
|  |  |  |  |  |  |  |  |  |  |  |  |  |  |  |  |  |  | |  | |  |
|  |  |  |  |  |  |  |  |  |  |  |  |  |  |  |  |  |  | |  | |  |
|  |  |  |  |  |  |  |  |  |  |  |  |  |  |  |  |  |  | |  | |  |
|  |  |  |  |  |  |  |  |  |  |  |  |  |  |  |  |  |  | |  | |  |
|  |  |  |  |  |  |  |  |  |  |  |  |  |  |  |  |  |  | |  | |  |
|  |  |  |  |  |  |  |  |  |  |  |  |  |  |  |  |  |  | |  | |  |
|  |  |  |  |  |  |  |  |  |  |  |  |  |  |  |  |  |  | |  | |  |
|  |  |  |  |  |  |  |  |  |  |  |  |  |  |  |  |  |  | |  | |  |
| *TSS* | | | | | *POM* | | | | | *SOM* | | | | |  | | | | | | |
| Se | 2 | 2.38 | 0.111 | 998 | Se | 2 | 4.35 | 0.014 | 997 | Se | 2 | 3.56 | 0.034 | 998 |  |  | |  | |  | |
| Si | 1 | 0.01 | 0.953 | 998 | Si | 1 | 0.56 | 0.457 | 995 | Si | 1 | 0.23 | 0.613 | 998 |  |  | |  | |  | |
| St(Si) | 6 | 1.48 | 0.215 | 999 | St(Si) | 6 | 2.04 | 0.075 | 998 | St(Si) | 6 | 1.40 | 0.210 | 998 |  |  | |  | |  | |
| Se×Si | 2 | 0.14 | 0.854 | 999 | Se×Si | 2 | 0.80 | 0.452 | 998 | Se×Si | 2 | 3.05 | 0.059 | 998 |  |  | |  | |  | |
| Se×St(Si) | 12 | 0.75 | 0.706 | 998 | Se×St(Si) | 12 | 0.73 | 0.705 | 996 | Se×St(Si) | 12 | 1.19 | 0.266 | 999 |  |  | |  | |  | |
| Residual | 46 |  |  |  | Residual | 46 |  |  |  | Residual | 198 |  |  |  |  |  | |  | |  | |
| Total | 69 |  |  |  | Total | 69 |  |  |  | Total | 221 |  |  |  |  |  | |  | |  | |
|  |  |  |  |  |  |  |  |  |  |  |  |  |  |  |  |  |  |  |  |  |  |
|  |  |  |  |  |  |  |  |  |  |  |  |  |  |  |  |  |  |  |  |  |  |

The significance (*p*) is based on the number of permutations listed in the final column. Significant results are taken at *p* ≤ 0.01 to account for multiple hypothesis tests and are underlined. Abbreviations are given in Table 1. Significant interactions and main effects were explored by *post-hoc* tests, using Monte Carlo approximate *p*-values [63] where insufficient unique permutations existed for meaningful tests. Results of significant post-hoc tests are described in the text, but are not tabulated.
