# Supplementary material for: Complex, Dynamic Combination of Physical, Chemical and Nutritional Variables Controls Spatio-Temporal Variation of Sandy Beach Community Structure
Source: PLoS One. 2011 Aug 17;6(8):e23724. doi: 10.1371/journal.pone.0023724 (PMC3157432; doi:10.1371/journal.pone.0023724)
Supplement: Table S3 — Two-way fixed-effects PERMANOVA on univariate and multivariate measures of community structure. (DOCX) [file pone.0023724.s005.docx]

Table S3. Two-way fixed-effects PERMANOVA on univariate and multivariate measures of community structure.

|  | **df** | **Pseudo-*F*** | ***p*** | **Perm** |  | **df** | **Pseudo-*F*** | ***p*** | **Perm** |
| --- | --- | --- | --- | --- | --- | --- | --- | --- | --- |
| *Species richness* |  |  |  |  | *Abundance* | | | | |
| Se | 2 | 8.89 | 0.004 | 999 | Se | 2 | 5.21 | 0.003 | 999 |
| Si | 1 | 6.32 | 0.012 | 992 | Si | 1 | 7.64 | 0.009 | 999 |
| St(Si) | 6 | 1.69 | 0.139 | 998 | St(Si) | 6 | 1.64 | 0.154 | 996 |
| SexSi | 2 | 7.72 | 0.001 | 999 | SexSi | 2 | 16.32 | 0.001 | 999 |
| SexSt(Si) | 12 | 1.96 | 0.065 | 999 | SexSt(Si) | 12 | 3.64 | 0.001 | 997 |
| Residual | 48 |  |  |  | Residual | 48 |  |  |  |
| Total | 71 |  |  |  | Total | 71 |  |  |  |
| *Community structure by transect* | | | | | *Community structure by station* | | | | |
| Se | 2 | 8.47 | 0.001 | 998 | Se | 2 | 3.14 | 0.003 | 999 |
| Si | 1 | 3.75 | 0.007 | 999 | Si | 1 | 1.61 | 0.161 | 999 |
| St(Si) | 6 | 1.00 | 0.510 | 999 | St(Si) | 6 | 0.62 | 0.923 | 998 |
| SexSi | 2 | 5.36 | 0.001 | 998 | SexSi | 2 | 1.13 | 0.362 | 998 |
| SexSt(Si) | 12 | 2.22 | 0.001 | 995 | Residual | 12 |  |  |  |
| Residual | 48 |  |  |  | Total | 23 |  |  |  |
| Total | 71 |  |  |  |  |  |  |  |  |

Statistics and abbreviations as per Tables 1 and 2. Underlined values indicate significant differences.
